# Urinary Microbiota—Are We Ready for Prime Time? A Literature Review of Study Methods’ Critical Steps in Avoiding Contamination and Minimizing Biased Results

**DOI:** 10.3390/diagnostics10060343

**Published:** 2020-05-27

**Authors:** Alin Adrian Cumpanas, Ovidiu Gabriel Bratu, Razvan Tiberiu Bardan, Ovidiu Catalin Ferician, Andrei Dragos Cumpanas, Florin George Horhat, Monica Licker, Catalin Pricop, Octavian Marius Cretu

**Affiliations:** 1 Department of Urology, “Victor Babes” University of Medicine and Pharmacy, 300041 Timisoara, Romania; cumpanas.alin@umft.ro (A.A.C.); bardan.razvanumft.ro@gmail.com (R.T.B.); ovidiu.ferician@yahoo.com (O.C.F.); 2 Department of Urology, Emergency Military Central Hospital, “Carol Davila” University of Medicine and Pharmacy, 020021 Bucharest, Romania; ovi78doc@yahoo.com; 3 Faculty of Medicine, “Victor Babes” University of Medicine and Pharmacy, 300041 Timisoara, Romania; 4 Department of Microbiology, “Victor Babes” University of Medicine and Pharmacy, 300041 Timisoara, Romania; horhat.florin@umft.ro (F.G.H.); licker.monica@umft.ro (M.L.); 5 Department of Urology, Gr.Tr.Popa University of Medicine and Pharmacy, 700115 Iasi, Romania; bobopricop@yahoo.com; 6 Department of Surgery, “Victor Babes” University of Medicine and Pharmacy, 300041 Timisoara, Romania; tavicretu@yahoo.com

**Keywords:** urinary microbiota, 16S mRNA gene sequencing, contamination

## Abstract

Within the last few years, there have been an increased number of clinical studies involving urinary microbiota. Low-biomass microbiome sequencing (e.g., urine, lung, placenta, blood) is easily biased by contamination or cross-contamination. So far, a few critical steps, from sampling urine to processing and analyzing, have been described (e.g., urine collection modality, sample volume size, snap freezing, negative controls usage, laboratory risks for contamination assessment, contamination of negative results reporting, exploration and discussion of the impact of contamination for the final results, etc.) We performed a literature search (Pubmed, Scopus and Embase) and reviewed the published articles related to urinary microbiome, evaluating how the aforementioned critical steps to obtain unbiased, reliable results have been taken or have been reported. We identified different urinary microbiome evaluation protocols, with non-homogenous reporting systems, which can make gathering results into consistent data for similar topics difficult and further burden the already so complex emerging field of urinary microbiome. We concluded that to ease the progress in this field, a joint approach from researchers, authors and publishers would be necessary in order to create mandatory reporting systems which would allow to recognize pitfalls and avoid compromising a promising field of research.

## 1. Introduction

The urinary tract was considered a sterile environment until the last decade when high throughput molecular DNA sequencing technologies demonstrated the contrary: even in the absence of urinary tract infection, the urine is not a sterile biofluid; it contains a variable microbial spectrum and the imbalance of the urinary microbiome is supposed to be involved in different urologic pathologies [1,2,3]. In-depth knowledge of urinary microbiota is expected to offer a better understanding of its metabolic, functional and networking aspects, to unveil the pathogenic pathways in urologic diseases and to further guide specific treatments [1].

The perspective of urinary microbiome as the “magic bullet” for the good health status of the urinary system and the opportunity to find correlations or to fathom its role in disease mechanisms led to a rapid shift in the number of studies aiming to identify urinary microbiome’s classes, orders, families, genera and even distinct species. Within the last decade, studies involving microbiome have tried to find correlations with cancer, diabetes, autism, urinary stone disease, prostate cancer, bladder cancer, etc. As W.P. Hanage mentioned, this emerging field—the microbiome—should be extremely meticulously and cautiously explored, being of paramount importance to unveil at least a few aspects: whether the current experiments detect differences that matter, to find the differences between causation and correlation and to find the mechanisms of action. He suggested to look dispassionately at the data. Otherwise, as “in pre-scientific times when something happened that people did not understand, they blamed spirits”, nowadays “we must resist the urge to transform our microbial passengers into modern-day phantoms” [4].

The microbiome is defined as the entire habitat of microorganisms (bacteria, archaea, lower eukaryotes, higher eukaryotes, and viruses), their genomes and the surrounding environmental conditions, whilst the microbiota is defined as the assemblage of microorganisms present in a defined environment [5].

Microbiome studies were initially focused on gut microbiota, thereafter followed by other high-volume microbiome organs, e.g., vagina, skin, mouth [6,7,8]. The obtained results were optimistic, revealing the importance of microbiota in maintaining homeostasis and the role of dysbiosis in disease pathogenesis [1]. The studies further extended to urinary, placenta and even blood microbiome, with only a few and yet inconsistent results [5,9,10,11,12].

There are two major types of microbiota: high volume microbiota (e.g., gut microbiota with around 10^11^ microbiome components per gram, vaginal microbiota, skin microbiota) and low-volume microbiota (e.g., urinary microbiota with less than 10^5^ microbiome components per mL in healthy population, lung microbiota) [13,14,15,16]. In these conditions, for an emerging area of research, a clear and well-defined methodology and technique should be established for every study in order to avoid biases and/or inconclusive results, which are more frequently expected compared with high-volume microbiota [1].

The metagenomic techniques (e.g., quantitative polymerase chain reaction (PCR) amplification of the 16S rRNA gene, shotgun sequencing) are extremely sensitive methods for low-volume microbiota. Meanwhile, they can easily detect contaminant DNA (DNA from sources other than the studied samples) or cross-contaminant DNA (DNA from other studied sample) from sampling to the DNA extraction and gene sequencing leading to biased results even in the last steps of bioinformatics data analysis [17].

The most important issues recommended to be resolved and reported in low-microbial-biomass microbiome studies in order to avoid biased results are: urine sampling (method of urine collection, urine volume, snap freezing in liquid nitrogen), laboratory environment, assessing DNA contamination (negative controls for sampling, DNA extraction and sequencing), determining the level of contamination by comparison to controls, as well as exploring contaminant taxa within each study and reporting their impact on the interpretation of the results [17].

In this article, we present the results of a review of the literature regarding the methodology of urinary microbiome studies. In other words, we reviewed how the essential steps of microbiome analysis methodology—described so far in the literature as prone to potential risks, controversies and pitfalls related to contamination and bias risks—have been implemented in order to avoid the potential biases within clinical studies as a prerequisite to fully relying on further microbiome-related answers to clinical questions.

## 2. Material and Methods

### 2.1. Evidence Acquisition

We performed a literature review based on three search sources: PubMED/Medline, Scopus and Embase from 2000 to January 2020, using medical subject headings (MeSH), query strings or Emtree vocabularies, respectively. “Urinary AND microbiome”, “urinary AND microbiota”, “urine AND microbiome”, “urine AND microbiota” and “urobiota” were used as search terms for the titles and abstracts to find the articles. The review process followed the statement guidelines of the Preferred Reporting Items for Systematic Reviews and Meta-Analysis (PRISMA) [18].

### 2.2. Study Selection and Data Extraction

Two reviewers independently evaluated the primary data and decided on the inclusion in the final analysis. For articles with divergent opinion, inclusion/exclusion from the final analysis has been chosen by consensus between all the authors. Articles in other languages than English, articles without an abstract, those without original data (comments, editorial reviews or reviews), or not evaluating human urinary microbiota, those without a detailed description of the technique/study method and those using techniques other than 16S rRNA PCR amplification of the bacterial DNA were excluded from the final analysis.

**Figure 1:**
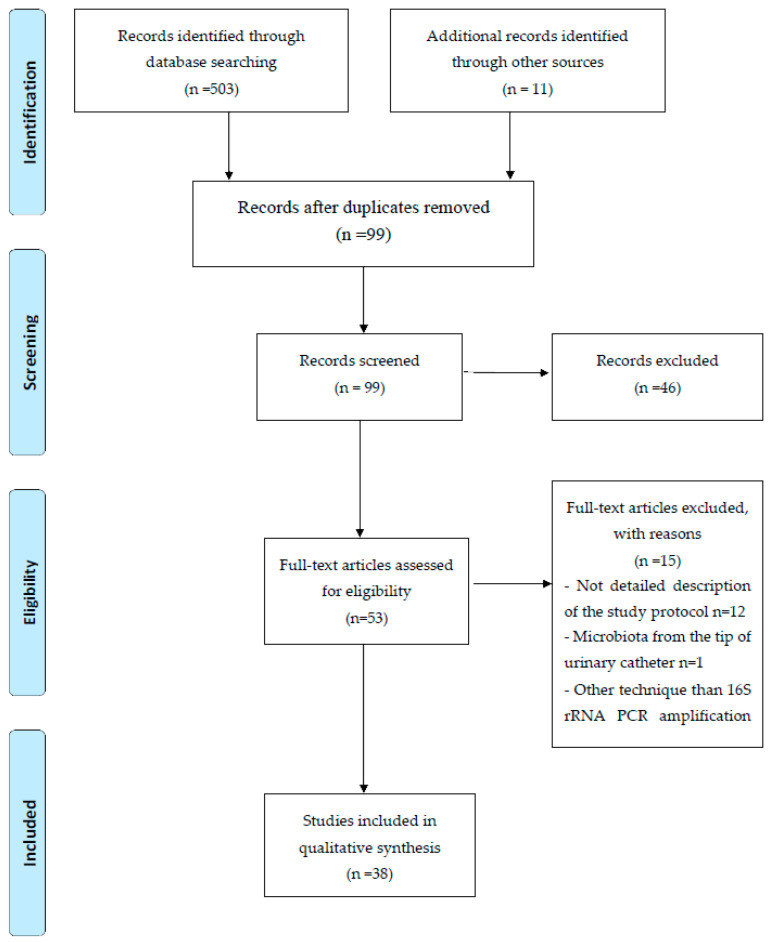
Reporting Items for Systematic Reviews and Meta-Analysis (PRISMA) flowchart of literature search.

The detailed flowchart of the literature search and study selection, as well as the inclusion and exclusion criteria according to PICOS items are presented below (Figure 1 and Table 1).

For data extraction we created a standardized Microsoft^®^ Excel format which was filled in by two authors according to the data from the selected articles. A third author reviewed the data before the final analysis.

## 3. Results

### 3.1. Study Characteristics

From the 38 studies included in this review, 33 out of 38 (86.8%) were single-center studies, while 5 out of 38 (13.2%) were multi-center studies. The majority of them were conducted in the USA (24/38—63.1%), the others having been performed in China (6/38—15.8%), Europe (5/38–13.1%), Australia (2/38—5.3%) and Africa (1/38—2.7%). A description of the study design is presented in Table 2. Twenty-five studies (25/38—65.7%) were controlled studies or cross-sectional studies, while 13 out of 38 (34.3%) were without a control group.

### 3.2. Population and Demographic Data

The total number of patients included in these studies was 3009, the majority of them being females (2170/3009—72.1%). Most of the studies included female patients only (15 studies out of 38—40.5%, with 1835 female patients included in these studies, representing 84.5% of the total female population from the studies). In 13 studies (13/38—33.5%) patients of both genders were included (691/3009—22%), whilst in 10 studies (10/38—26%) only males (483 patients) were included.

### 3.3. Studied Correlations with Different Pathologies

The relations of urinary microbiome with different pathologies, conditions, symptoms, procedures were studied as follows: urinary incontinence/urge urinary incontinence/mixed urinary incontinence (six studies), interstitial cystitis (2 cases), kidney stones (3 studies), lower urinary tract symptoms (LUTS) (2 studies), kidney transplant/graft function (5 studies), overactive bladder (2 studies), bladder cancer (3 studies), prostate cancer/prostate biopsy (3 studies), bacterial vaginosis (1 study), chronic prostatitis (2 studies), hematuria (1 study), bladder schistosomiasis (1 study), in vitro fertilization (1 study), bladder urinary oxygen tension (1 study), oral probiotics consumption (1 study), urine sampling method (1 study), pediatric gut microbiome (1 study), as well as the evaluation of urinary microbiome in the healthy population (2 cases).

### 3.4. Urine Sampling

In our review, in only 13 out of 28 studies with female patients (46%), adding up to a total of 963 females (44.3% of female patients), the urine was collected by urethrovesical catheterization, while in 15 out of 28 studies (55.7%) (1207/2170 female patients—55.7%) the urine was not collected by urethrovesical catheterization, mid-urinary stream being the method of choice.

The protocol for collected urine volume was not reported in six studies involving 542 patients (18%); it was less than 30 mL in seven studies (708 patients—23.5%), and it was between 30–50 mL in 25 studies (1759 patients—58.4%).

The snap freezing of the urine at −80 °C within less than four hours from sampling was reported as part of the study protocol in 23 studies (60%) involving 1645 patients (54.6%), while in 14 studies (1272 patients—42.2%) snap freezing was performed after more than four hours from sampling. In two studies (92 patients—3%) snap freezing was not performed at all.

### 3.5. Mitigating the Impact of Contamination during DNA Extraction, Sequencing and Data Analysis

All 38 studies were conducted in laboratories with expertise in DNA sequencing. Thus, it is reasonable to assume that all of them meet the requirements related to the laboratory environment preparation to minimize specimen contamination.

The reported negative controls (negative control sampling, negative control DNA extraction and negative control no-template amplification) within the study protocols were as follows:

Three negative controls were reported in 11 studies (1070 patients—35.6%) [20,25,26,36,40,45,47,48,50,52].

Two negative controls were used in 11 studies (885 patients—29%) [19,29,30,31,32,34,35,42,44,53,56].

One negative control was used in two studies (73 patients—2.9%) [39,41,55].

Negative controls were not reported in 15 studies (981 patients—32.5%) [21,24,27,28,33,37,38,43,46,49,51,54].

The hypervariable region targeted by the primer was V4 solely in 17 studies [20,22,30,32,33,34,35,38,40,41,42,43,45,48,49,55,56]; V3 in one study [28]; V6 in two studies [31,44]; V9 in one study [27]; V3–V4 in four studies [21,37,50,54]; V1–V3 in one study [19]; V1–V2 in one study [29]; V3–V5 in one study [39]; V4–V6 in one study [36]. One study used primers targeting V2, 4, 8 and V3, 6, 7, 9 regions [24], and one study targeted V1–3 and V4–6 regions [26].

In eight studies (21%), the level of contamination comparing samples to control was discussed in the article [20,28,29,34,35,36,44,55]. In the other articles, this issue was not discussed. Contamination was further explored in six studies (15.7%) [20,29,34,36,44,55], while the impact of contamination on the results and the possible biased results was discussed in four studies (10.5%) [29,34,44,55].

## 4. Discussion

In this literature review, we found that for the majority of the studies female patients were involved (72.1%), the preferred study focus being urinary incontinence and kidney transplant patients’ graft function. However, out of 38 studies, there are 18 study themes, confirming the previous reported data that there is an increasing interest in studying the relationship between the microbiome and almost all medical pathology [57,58]. We identified a range of study protocols and a paucity of data on specific topics which makes a meta-analysis of results quite impossible. Urinary microbiome studies are usually trying to identify genera, recently focusing on distinct species characterization. Although the current techniques offer mainly a qualitative/semi quantitative evaluation of the urinary microbiome, the bioinformatics software analysis of alpha diversity (diversity within individuals) and beta diversity (dissimilarities between individuals) already offers data which are hard to clinically interpret. As an example, we did not identify in the literature a “core” urinary microbiome present in the healthy population, from where further study analysis could proceed. It is clear that in the coming years, the integration of new, translational microbiota methods in medicine will lead to a kind of “clinical microbiota expert” who would be able to gather information and data from a specific field as an essential step to interpret results, to define causality relations and to identify the mechanisms of action for further clinical and therapeutic approaches [59].

Urine sampling is a crucial part of the microbiota study, due to the scarcity of the urinary microbiome. The key principle is to collect the urine directly from the bladder, in order to avoid the contamination from the proximity high-volume microbiota environments (e.g., vagina or skin). There are three methods to collect the urine:

(1) clean-catch midstream urine specimen—the most frequent urine sampling method is suitable for men due to the lower risk of contamination comparing with those of women (vaginal and skin microbiota). However, when using this method, even in men, there is a risk of obtaining a mixture of bladder and urethral microbiota, thus the term genitourinary microbiota should be used when analyzing samples collected this way [1]. In the meantime, it seems that this method allows no contamination with microbiota from coronal sulcus [49,60].
(2) suprapubic aspiration—is considered the most reliable and the cleanest method of sampling, with the lowest risk of contamination. The only limitation is that it is an invasive method which can lead, rarely, to bowel perforation or bleeding [61].
(3) urethrovesical catheterization—offers a good quality urine sample, especially in women, without being an invasive method like the suprapubic aspiration. Meanwhile it offers a lower number of bacteria than midstream urine specimens [1]. Studies comparing urethrovesical catheterization with suprapubic aspiration in women showed similar microbiome composition [49,61].

So far, all three above-mentioned methods are considered reliable sampling methods for men whilst for women only urethrovesical catheterization or suprapubic aspiration offer urine that is qualitative enough for analysis. In this review, we found that 44.3% had the urine collected by urethrovesical catheterization, while 55.6% had the urine collected from mid-urinary stream, predisposing to potential biased results from the very beginning of the study.

Sample urinary volume is another key issue for a good quality urinary microbiome research field. A 30–50 mL sample of urine is considered optimal for urinary microbiota analysis if the urine was collected by urethrovesical catheterization, offering a 95% success rate [22]. For voided urine, 1–2 mL was reported to be enough to characterize the microbiome (85% success rate) [56]. Our data reveals that in 58.4% of patients a volume of 30–50 mL of urine was collected. None of the studies reported less than 30 mL for catheter-collected urine, thus, theoretically this issue should not have been a bias factor.

Although there is no consensus regarding the sample storage conditions, immediate snap freezing at −80 °C in liquid nitrogen is usually recommended, due to the risk of alteration on urine microbiota composition if the freezing process has been delayed (due to exposure to oxygen, to UV light, storage components, osmotic stress, etc.). However, due to the lack of data regarding the influence of storage conditions on urinary microbiota, this could be seen as an anecdotical assumption, based on the low-biomass volume of urinary microbiota and the regular urine culture storage recommendations. So far, vaginal, gut and skin microbiota studies have not revealed a significant influence of storage on microbiota composition [1,10,62].

We identified the snap freezing of the urine at −80 °C within less than four hours from sampling in 60% of the studies involving 54.6% of patients, while in 14 studies (42.2% patients) snap freezing was performed later than four hours from sampling. In two studies (3%), snap freezing was not reported. Thus, a bias factor can emerge from this approach until further specific data for urinary samples storage for urinary microbiota analysis becomes available.

Once the sample arrives in the lab, the first steps should ensure that the risk of DNA contamination/cross-contamination is minimized. Any DNA from reagents, free label consumables, laboratory instruments, as well as human DNA from the researchers can contaminate the samples. Cross-contamination is defined as the accidental exchange of DNA between the samples in any phase of a study. For this reason, the samples should be handled in the cleanest, most low-contaminant, most isolated environment; the personnel should be protected (gloves, masks, etc.) It is recommended that the lab where the samples are handled should have previously been treated with UV radiation and for the surfaces to have been recently treated with sodium hypochlorite solution in order to minimize potential contaminant DNA from the lab environment [63]. Acknowledging that even DNA-free labeled consumables, as well as tubes and pipettes can contain degraded microbial DNA, ethylene oxide treatment (for surfaces) and UV light exposure (for reagents) are recommended to avoid DNA contamination [63,64,65]. Molecular biology grade water, PCR reagents and DNA extraction kits can be potential sources of contamination [66,67,68,69]. For these reasons, even when strict internal control protocols are fulfilled, it is recommended to use reagents, extraction kits, etc., from the same batches. Moreover, it is recommended to use a lab with a strictly controlled environment, physically isolated from post-PCR facilities [17].

All studies included in our review were conducted in cooperation with laboratories with expertise in DNA sequencing. Thus, although not specifically mentioned in the articles—probably due to article length constraints—it can be assumed that all laboratories took all necessary precautions and they approached the low-biomass microbiome research projects with the goal of avoiding data misinterpretation by taking all the necessary measures in order to minimize the contamination and cross-contamination from sampling to processing and data analysis.

There are authors suggesting that three types of negative controls should be used: sampling blank controls, DNA extraction blank controls and no-template PCR amplification controls. Usually at least two, up to eight negative controls (in case of larger studies using robotic systems) are recommended [1,17,70]. In our review, 21 studies met these requirements, having two or three negative controls (64.9%). However, in 15 studies negative controls were not reported as part of the study protocol (32.6%), while in two studies only one negative control was reported. It is worth mentioning that the negative sampling control (no urine in collecting recipient) was the least used negative control (it was not used in 24/38 studies), although collecting a sample within an inappropriate environment, with inappropriate equipment, etc., can significantly contaminate the probe. The lack of a negative control makes the identification of this pitfall quite impossible [63].

For DNA extraction, the use of DNA extraction blank control is a recommended and useful tool, as it can reveal accidental contamination [17]. There are authors suggesting that for low-biomass microbiome samples (urine, blood, lungs), there is a critical tipping point where contaminant DNA becomes dominant in sequencing, the internal control (DNA extraction blank control) being of paramount importance [9]. There are solutions described in the literature which are able to target and remove vertebrate DNA by binding the methylated CpG islands (specific for eukaryotes and extremely rare in bacterial DNA), impeding the PCR amplification of contaminant DNA [71].

However, it should be mentioned that, besides the risk of contamination or cross-contamination which could be avoided, the chosen method for bacterial wall lysis and for DNA purification is of paramount importance: if the lysis method is too weak, some bacteria with thick cellular walls will remain intact, thus, their DNA will not be sequenced; by contrast, a rough method of bacterial wall lysis/DNA purification risks to compromise DNA integrity. It is worth noting that there is a paucity of studies related to the optimal methods of bacterial cell lysis and DNA purification for low-microbial-biomass samples (such as urine, blood, lungs), in contrast with high-microbial-biomass samples (gut microbiome) which have better defined protocols. There are studies suggesting that for specific microbial taxa, specific DNA extraction protocols should be used to minimize biases [10,72,73].

PCR amplification/gene sequencing follows the DNA extraction and involves the 16S rRNA gene amplification. The 16S rRNA gene is a well-conserved gene, which exists in all bacterial DNA. The gene has multiple hypervariable regions defining species, interposed between common, highly conserved genetic regions. The common, conserved regions are used to be targeted by primers for PCR amplification of the bacterial DNA. The hypervariable region allows taxonomic classification of the bacteria. Bacterial DNA extraction, PCR amplification of the 16S rRNA gene and DNA sequencing are the main steps in microbiota studies. One should know that the 16S rRNA sequencing does not make the difference between viable, non-viable bacteria and even free-cell DNA, thus, the method offers a snapshot of viable and non-viable bacteria, every single extrinsic/contaminant DNA being a potential source of misleading results [1].

Although the 16S rRNA gene is a remarkably well-conserved one, the PCR amplification technique for low-volume biomass microbiota can bring potential bias factors, causing misleading study results. The most commonly cited sources of PCR amplification/gene sequencing biases are: the DNA polymerase fidelity, the bacterial DNA quantity, the PCR cycle numbers and the primers affinity for the hypervariable region of the 16S rRNA of different bacteria [1,74]. Out of these, the latter two are the most commonly cited as bias factors: the number of PCR cycles and the primer/targeted region.

The higher the number of PCR cycles, the more statistically significant is the risk of amplifying contaminant DNA or obtaining chimeric sequences through two distinct DNA anneals forming a new alien sequence [1]. However, as chimeric sequences can be recognized and removed by software algorithms, it should not be considered a potential bias factor as long as bioinformatics software is used.

The second bias factor in PCR amplification is the choice of primer and targeted hypervariable region. The primer targeting the hypervariable region could have a higher affinity for certain bacteria and a lower one for the others, which could mislead the PCR amplification results and subsequent interpretation. As there is a paucity of data regarding the hypervariable regions of urinary microbiome bacteria, there are different approaches, with most researchers using primers for V4, V1–V3 and less frequently for V1–V2 and V6 hypervariable region [1,12,19,29], all of them acknowledging that it could miss or underevaluate certain species of bacteria. It is reasonable to expect within the next years, as more in-depth research will be done and more precise results will be needed in urinary microbiome evaluation, to see panels of primers which would complementarily target different hypervariable regions, making possible the characterization of microbiota up to the species level.

The most used primer identified in this review was for the V4 region (17 studies), V3–V4 in four studies. Two studies used an extensively modern targeting approach for V2, 4, 8 and V3, 6, 7, 9 regions [24] V1–3 and for V4–6 regions [26].

Once the PCR amplification is complete, gene sequencing is the next step. The unique bacterial DNA gene sequence is used for taxonomic classification. There are different sequencing technologies, yielding excellent results, for urinary microbiota, all of them being highly dependent upon the quality of the aforementioned steps, from urine collection to PCR amplification. Sequencing errors, usually base substitutions, insertions, deletions or substitution miscall can be recognized and corrected by correction sequencing tools/techniques (e.g., quality trimming, denoising algorithms, sequence replicates corrections) [1,75].

The obtained raw sequencing data should be further processed and interpreted. The workflow of this process is a complex one; it involves bioinformatics and is variable from one center of research to another. The computational analysis involves removing the primer sequence, separating the sequences from multiple samples (demultiplexing), chimeric removal, removing the low-quality sequences, applying scores/algorithms in order to avoid poor quality sequences to downstream analysis [1]. As the bioinformatics software continuously changes and improves, there are substantial approach differences, the details of this process exceeding the scope of this paper.

Thereafter, the sequences are grouped by similarity using clustering methods (OTU—operational taxonomic unit) or Amplicon Sequence Variant Identification (ASV) algorithms. Recently, OTU clustering was cited as leading to an overestimation of the number of bacteria and thus, to the incorrect evaluation of bacterial diversity in low-mass microbiota [1].

The taxonomic assignment (phylum, class, order, family, genus and species) follows the sequences grouping, using bioinformatic databases for taxonomic assignment and algorithms.

Interestingly, in eight studies only (21%) the level of contamination was discussed, the contamination was further explored in comparison with controls in only six studies and its impact on the final results was discussed in only four studies. This leads to the lack of reports regarding the contaminant species, their impact on final results and it could sometimes interfere with the correct interpretation of results.

We acknowledge that sometimes there are length limitations to the article, which tend to shorten the study methods description to essential steps and to not describe all the steps (which could be extremely important for other researchers in study protocol evaluation and data reliability). On the other hand, we should admit that for the emerging field of low-biomass microbiota there is a huge need of high-quality, extremely well-conducted studies and study protocols, with minimal biases. Assuming these facts, it would probably be a good approach for researchers, authors and publishers to create and to adhere to minimal standards checklists, as is that suggested by Eisenhofer [17] (the RIDE criteria) which standardize the low-biomass microbiome study reports. By this approach, we would be able to collect larger, significant, reliable and unbiased data which could be useful to move forward from rising questions and hypotheses (sometimes received with a healthy dose of skepticism) to undoubted, scientifically proven answers to many unanswered issues related to urinary microbiota and different pathologies.

## 5. Conclusions

Within recent years, there have been an increasing number of clinical studies related to urinary microbiota. As every emerging field, many hypotheses have been raised and need to be confirmed or refuted by clinical data. Being easily exposed to contamination, the study of urinary microbiota needs well-designed and extremely well-conducted study protocols. For a fast forward movement in this research field, in order to avoid losing valuable data, to avoid having possible high quality data being received with skepticism and to be able to gather similar data without the risk of biasing results, it is essential for researchers, authors and publishers to define mandatory checklists for study protocol reporting as prerequisites of publishing in scientific journals.

## Figures and Tables

**Table 1:** The inclusion and exclusion criteria according to PICOS.

PICO Element	Inclusion	Exclusion
**Population**	All human subjects	Animal studies
**Intervention**	Urinary microbiome evaluated using 16S rRNA PCR amplification of the bacterial DNA technique	Any other technique used for urinary microbiota
**Comparisons**	No comparisons of methodology	
**Outcomes**	Detailed presentation of the technique’s steps	Detailed technique of 16S rRNA PCR amplification of the bacterial DNA not presented in the article’s full text
**Types of the study**	Controlled and non-controlled Original data only	Anything other than original articles (reviews, comments, editorial reviews, case reports) Articles published before 2000
**Times and settings**	Any time Any setting	

**Table 2:** Study design, settings, population included and study focus in relation with microbiome (CPPS—chronic pelvic pain syndrome, UTI—urinary tract infection, LUTS—lower urinary tract symptoms, HTN—arterial hypertension, AKI—acute kidney injury).

Author	Study Focus Related to Microbiome	N	Males	Females	Single Site/Multicentric + Country	Controlled/Non-Controlled
Lewis, 2013 [19]	Healthy population	16	6	10	Single center—UK	Non-controlled
Pearce, 2015 [20]	Women with urge urinary incontinence	182	0	182	Single center—USA	Non-controlled
Shoskes, 2016 [21]	Chronic prostatitis, CPPS	50	50	0	Single center—USA	Controlled
Karstens,2016 [22]	Urge incontinence	20	0	20	Single center—USA	Controlled
Abernethy, 2017 [23]	Interstitial cystitis	40	0	40	Single center—USA	Controlled
Modena, 2017 [24]	Kidney transplant patients	48	30	18	Single center—USA	Controlled
Rani, 2017 [25]	Kidney transplant patients	29	18	11	Single center—USA	Controlled
Komesu, 2017 [26]	Urinary incontinence	210	0	210	Multicenter—USA	Controlled
Curtiss,2017 [27]	Overactive bladder	98	0	98	Single center—UK	Controlled
Adebayo, 2017 [28]	Urogenital schistosomiasis	70	36	34	Single center—Nigeria	Non-controlled
Gotschick, 2017 [29]	Bacterial vaginosis	123	31	92	Single center—Germany	Controlled
Wu, 2017 [30]	Overactive bladder and psychological factors	55	0	55	Single center—China	Controlled
Shrestha, 2018 [31]	Males with/without biopsy for diagnosis of prostate cancer	135	135	0	Single center—USA	Controlled
Wu, 2017 [32]	Bladder cancer	60	60	0	Single center—China	Controlled
Chen, 2018 [33]	Urge incontinence/detrusor overactivity and recurrent UTI	39	0	39	Single center—Australia	Non-controlled
Fok, 2018 [34]	LUTS on females	126	0	126	Single center—USA	Non-controlled
Wu, 2018 [35]	Kidney transplant graft dysfunction	67	19	48	Multicenter—USA	Controlled
Komesu, 2018 [36]	Mixed urinary incontinence	212	0	212	Multicenter—USA	Controlled
Koedooder, 2018 [37]	In vitro fertilization	350	0	350	Single center—The Netherlands	Controlled
Mai, 2019 [38]	Bladder cancer	24	18	6	Single center—China	Non-controlled
Alanee, 2019 [39]	Prostate cancer	30	30	0	Single center—USA	Controlled
Dornbier, 2019 [40]	Urinary calcium stones	52	23	29	Single center—USA	Non-controlled
Moynihan, 2019 [41]	Hematuria/tobacco smoke	43	43	0	Single center—USA	Non-controlled
Shannon, 2019 [42]	Bladder urinary oxygen tension	34	0	34	Single center—USA	Non-controlled
Alanee, 2019 [43]	Transrectal biopsy	30	30	0	Single center—USA	Controlled
Kassiri, 2019 [44]	Relation between pediatric gastrointestinal and urinary microbiome	10	10	0	Single center—USA	Controlled
Bresler, 2019 [45]	Interstitial cystitis	41	0	41	Single center—USA	Controlled
Wolff, 2019 [46]	Oral probiotics for UTI	7	0	7	Single center—USA	Controlled
Colas, 2020 [47]	Kidney transplant	98	59	39	Single center—France	Controlled
Price, 2020 [48]	Continent adult women	224	0	224	Single center—USA	Non-controlled
Pohl, 2020 [49]	Differentiation between urine collection method	20	14	6	Single center—USA	Non-controlled
Hourigan, 2020 [50]	Bladder cancer—urine collection: midstream vs. cystoscopy	22	14	8	Single center—USA	Non-controlled
Xie, 2020 [51]	Calcium kidney stones	43	43	0	Single center—China	Non-controlled
Bajic, 2020 [52]	Male LUTS with/without surgery	49	49	0	Single center—USA	Controlled
Liu, 2020 [53]	Kidney stone and HTN (pelvis urine)	62	42	20	Single center—China	Controlled
Gerges-Knafl, 2020 [54]	Transplant vs. non-transplant AKI	30	16	14	Single center—Australia	Controlled
Wu, 2020 [55]	Chronic prostatitis	63	63	0	Single center—China	Controlled
Thomas -White, 2016 [56]	Stress urinary incontinence	197	0	197	Multicentric—USA	Controlled
